# Backcalculating the Incidence of Infection with COVID-19 on the Diamond Princess

**DOI:** 10.3390/jcm9030657

**Published:** 2020-02-29

**Authors:** Hiroshi Nishiura

**Affiliations:** 1 Graduate School of Medicine, Hokkaido University, Kita 15 Jo Nishi 7 Chome, Kita-ku, Sapporo-shi, Hokkaido 060-8638, Japan; nishiurah@med.hokudai.ac.jp; Tel.: +81-11-706-5066; 2 Core Research for Evolutional Science and Technology (CREST), Japan Science and Technology Agency, Honcho 4-1-8, Kawaguchi, Saitama 332-0012, Japan

**Keywords:** incidence, forecasting, virus, statistical estimation, emerging infectious diseases

## Abstract

To understand the time-dependent risk of infection on a cruise ship, the Diamond Princess, I estimated the incidence of infection with novel coronavirus (COVID-19). The epidemic curve of a total of 199 confirmed cases was drawn, classifying individuals into passengers with and without close contact and crew members. A backcalculation method was employed to estimate the incidence of infection. The peak time of infection was seen for the time period from 2 to 4 February 2020, and the incidence has abruptly declined afterwards. The estimated number of new infections among passengers without close contact was very small from 5 February on which a movement restriction policy was imposed. Without the intervention from 5 February, it was predicted that the cumulative incidence with and without close contact would have been as large as 1373 (95% CI: 570, 2176) and 766 (95% CI: 587, 946) cases, respectively, while these were kept to be 102 and 47 cases, respectively. Based on an analysis of illness onset data on board, the risk of infection among passengers without close contact was considered to be very limited. Movement restriction greatly reduced the number of infections from 5 February onwards.

## 1. Introduction

An outbreak of novel coronavirus disease (COVID-19) has occurred on a cruise ship, the Diamond Princess [1]. The primary case remains unknown, but the index case, defined as the first identified case, is a passenger who started coughing from 19 January 2020 on board, disembarking the ship in Hong Kong on 25 January. As the case was diagnosed on 1 February, the ship was requested to remain in the ocean near Yokohama from 3 February onwards. Subsequently, the movement of all passengers was restricted on board from 5 February, for a matter of 14 days of quarantine. Out of a total of 3711 persons (consisting of 2666 passengers and 1045 crew members), 199 symptomatic cases have been diagnosed on board as of 24 February, and additional asymptomatic infections and symptomatic cases after disembarkation have also been reported.

One of the critical issues in infectious disease epidemiology is that the time of infection event is seldom directly observable. For this reason, the time of infection needs to be statistically estimated, employing a backcalculation method [2]. Using a sophisticated statistical model with doubly interval-censored likelihood and right truncation with an exponential growth of cases, the mean incubation period has been estimated to be about 5.0 days [3]. To understand the time-dependent risk of infection throughout the course of outbreak and estimate the effectiveness of the quarantine measure from 5 to 19 February 2020, I aimed to estimate the incidence of infection with COVID-19 and also predict the likely number of infections prevented by the quarantine measure.

## 2. Backcalculation and Forecasting

I analyzed the epidemic curve, *c*_t_, on day *t*, illustrated by the number of confirmed cases by the date of illness onset. The confirmatory diagnosis was made, using the reverse transcriptase polymerase chain reaction (RT-PCR). The date of illness onset was defined as the first date of fever. In addition to the date of illness onset, cases were classified by contact history inside the cabin and also by the type of membership, i.e., crew or passenger. Close contact was defined as having at least one cabinmate who was confirmed by RT-PCR.

We estimate the number of cases by time of infection, *i*_t_. Using the probability mass function of the incubation period of length *s*, *f*_s_, the incidence of infection is known to satisfy

(1) $$E\left(c_{t}\right)=\overset{t-1}{\underset{s=1}{\sum }}i_{t-s}f_{s},$$

where *E*(.) represents the expected value. As for *f*_s_, it is known that the mean and standard deviation are 5.0 and 3.0 days, respectively, best fitted by lognormal distribution [3]. Employing a step function, the incidence of infection was statistically estimated via a maximum likelihood method. The estimation was implemented independently by the history of contact and type of membership.

Regarding the real-time forecasting, we employed the so-called Richards model, an analogue to the generalized logistic model [4,5]:

(2) $$E\left(C_{t}\right)=\frac{Z}{\left(1+s\exp(-a(t-t_{i}\right){)}^{\frac{1}{s}}},$$

where $C_{t}$ is the cumulative incidence on day *t*, *Z* is the cumulative incidence at the end of the outbreak, *s* is the parameter that governs the flexibility of the logistic curve, *a* is the early growth rate of cases and *t*_i_ is the inflection point of the cumulative incidence curve. Assuming that the cumulative incidence is Gaussian distributed, four unknown parameters were estimated. The Richards model was fitted to two different datasets, i.e., (i) the dataset of the entire course of the epidemic and (ii) the dataset by 4 February 2020. The latter dataset corresponds to the time period without any impact of movement restriction that was in place from 5 February onwards.

## 3. Estimated Incidence

**Figure 1:**
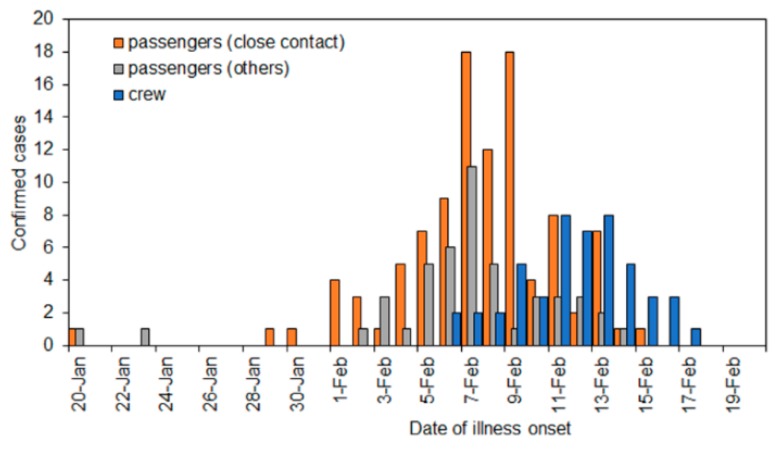
The number of confirmed cases of COVID-19 by contact history and the type of membership from 20 January to 20 February 2020 on the Diamond Princess (*n* = 199). Close contact was defined as passengers with a confirmed case among their cabinmates.

Figure 1 shows the epidemic curve by contact history and type of membership. The highest incidence of illness onset was observed on 7 February. The epidemic curve in a latter half period was dominated by crew members whose movement was not strictly controlled due to the need to continue service on the ship. The second dominating group was passengers with close contact history. The last illness onset date on board of a passenger without close contact was on 14 February.

**Figure 2:**
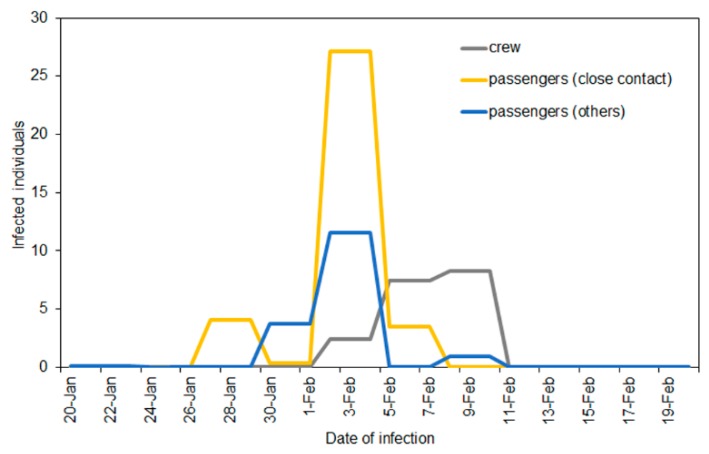
Estimated incidence of infection COVID-19 by contact history and the type of membership from 20 January to 20 February 2020 on the Diamond Princess (*n* = 199). Close contact was defined as passengers with a confirmed case among their cabinmates.

Estimating the incidence of infection, the peak incidence was identified for the period from 2 to 4 February among passengers both with and without close contact (Figure 2). The incidence of infection abruptly dropped after 5 February, the date of movement restriction. Among passengers without close contact, the incidence was estimated to be zero, except for 8–10 February 2020, during which 0.98 persons (95% confidence intervals (CI): 0, 7.74) per day were estimated to have been infected. The epidemic peak among crew members was seen for the period from 8 to 10 February 2020.

**Figure 3:**
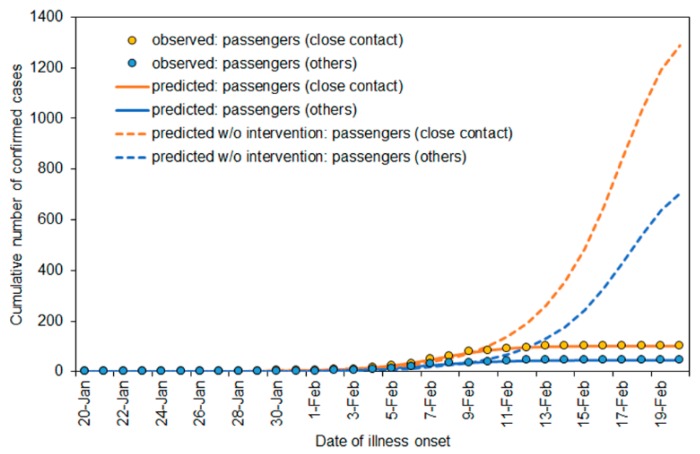
Comparisons between observed and predicted cumulative incidence of cases with COVID-19 on the Diamond Princess. The intervention, i.e., movement restriction, was in place from 5 February onwards. Dashed lines represent predictions without accounting for the dataset from 5 February 2020. Close contact was defined as passengers with a confirmed case among their cabinmates.

Figure 3 compares the cumulative incidence with and without movement restriction policy from 5 February. In the presence of intervention, the cumulative incidence among passengers with and without close contact and crew members were 102, 47 and 48 cases, respectively, as of 24 February 2020. These were well realized by the Richards model. Without intervention from 5 February onwards, it was predicted that the cumulative incidence with and without close contact would have been 1373 (95% CI: 570, 2176) and 766 (95% CI: 587, 946) cases, respectively.

## 4. Discussion and Conclusions

A large outbreak of COVID-19 occurred on a cruise ship. Estimating the incidence, the peak time of infection was shown to have been from 2 to 4 February, and the incidence abruptly declined afterwards. The estimated number of new infections among passengers without close contact was very small from 5 February, on which the movement restriction policy was imposed, and at most there was, on average, one case of infection per day from 8 to 10 February. Other than continued exposure among crew members, the estimated incidence in this study indicates that the movement restriction policy from 5 February 2020 was highly successful in greatly reducing the number of secondary transmissions on board. Based on an analysis of illness onset data on board (and before the disembarkation of a large number of passengers), the risk of infection among passengers without close contact was considered to be very limited

Among disembarked passengers, symptomatic cases have started to be reported on the ground in and outside of Japan. In particular, cases arising from passengers without close contact indicate a possible pathway of infection via mechanisms that were not covered by the abovementioned analysis that relied on symptomatic cases. Although the transmission via direct human-to-human contact was prevented by movement restrictions, the role of other modes of transmission, e.g., environmental and asymptomatic transmissions, should be further explored.
